# Zen Mountains: An Illusion of Perceptual Transparency

**DOI:** 10.1068/i0723sas

**Published:** 2015-04-01

**Authors:** Susan G. Wardle, Thomas A. Carlson

**Affiliations:** Department of Cognitive Science and ARC Centre for Cognition and its Disorders, Macquarie University, Sydney, Australia; Department of Cognitive Science and ARC Centre for Cognition and its Disorders, Macquarie University, Sydney, Australia

**Keywords:** depth perception, Gestalt principle of good continuation, image segmentation, nonreversing X-junction, perceptual grouping, transparency, visual illusion

## Abstract

The human visual system is usually very successful in segmenting complex natural scenes. During a trip to the Nepalese Himalayas, we observed an impossible example of Nature's beauty: “transparent” mountains. The scene is captured in a photograph in which a pair of mountain peaks viewed in the far distance appear to be transparent. This illusion results from a fortuitous combination of lighting and scene conditions, which induce an erroneous integration of multiple segmentation cues. The illusion unites three classic principles of visual perception: Metelli's constraints for perceptual transparency, the Gestalt principle of good continuation, and depth from contrast and atmospheric scattering. This real-world “failure” of scene segmentation reinforces how ingeniously the human visual system typically integrates complex sources of perceptual information using heuristics based on likelihood as shortcuts to veridical perception.

*“At first, I saw mountains as mountains and rivers as rivers*.

*Then, I saw mountains were not mountains and rivers were not rivers*.

*Finally, I see mountains again as mountains, and rivers again as rivers.*”

—Zen proverb

The Himalayas of Nepal are strikingly beautiful. Take a look at the photograph in Figure 1. Clear all thoughts from your mind and stare into the distance to observe the far-away peaks. If you persist and reach a Zen-like state, you may observe that the mountains look even more mysterious than at first glance. The two most distant peaks appear transparent. At first, you may think that you have achieved the ability to see through impenetrable solid rock through your mastery of a Zen-like state. Fortunately, decades of perception research can provide a more scientific explanation. The transparent mountains are highlighted in the boxed region of Figure 2a, and in the illusion both their segmentation and relative depth are misperceived. In the illusory interpretation, the nearer mountain appears to be defined by areas *y* and z, with region z overlapping with the more distant mountain defined by areas *x* and *y* (Figure 2a). The enlargement in Figure 2b clearly shows that this interpretation of the image is incorrect: in fact, region *z* belongs to the nearer mountain, and the more distant mountain is defined by areas *x* and *y*.

Three classic principles of visual perception underlie this illusion. First, the photograph is a nice example of contrast as a depth cue due to atmospheric scattering. Distant objects are viewed through more of the atmosphere than nearer objects, and hence have lower contrast. This is known as “aerial (or atmospheric) perspective” and psychophysical experiments have demonstrated that contrast is used as a pictorial depth cue by human observers (O'Shea, Blackburn, & Ono, 1994; Rohaly & Wilson, 1999). The closer mountains in Figure 1 have much greater contrast with the light background sky overhead than the more distant mountains. The relative difference in luminance between the distant mountain peaks produced by atmospheric scattering is one ingredient for the illusion of transparency in this scene.

**Figure 1.**
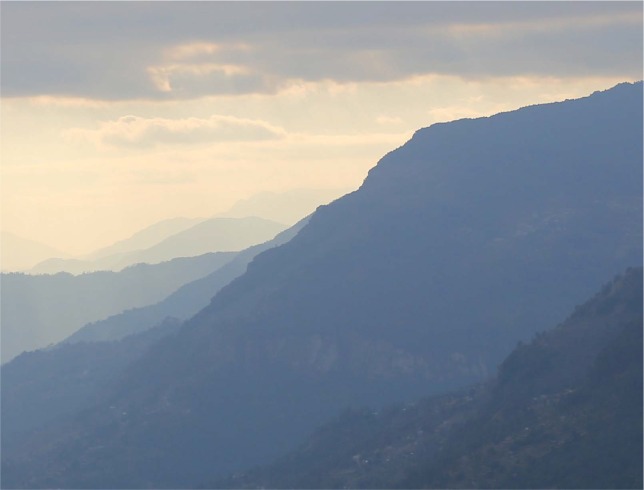
Transparent mountains in the Nepalese Himalayas (most distant peaks). [Photo: Thomas Carlson, Canon EOS 6D].

Metelli (1974) recognized that perceptual transparency is possible in the absence of physical transparency if opaque surfaces obey certain luminance conditions. The differences in relative luminance between the distant mountain peaks that are the subject of the illusion in Figure 1 are consistent with Metelli's (1974) constraints for perceptual transparency. The overlap between two transparent objects must be darker than either object itself. The two mountains in Figure 2a obey this rule, because they differ in luminance as a consequence of aerial perspective. The luminance of the overlap region (*z*) is consistent with a combination of the lighter luminances of areas *x* and *y.* This makes it plausible that areas *y* and *z* belong to the same (nearer) mountain, with region *z* marking the area of transparent overlap with a more distant mountain defined by area *x*. The four-region pattern produced by the alignment of contours defining regions *x, y*, and *z* (captured in the box inset in Figure 2a) is known as a *nonreversing X-junction* (Adelson & Anandan 1990; Anderson 1997). The relative luminances of the four areas retain their sign (i.e., their contrast polarity) across the X-junction, because *w*<*x* and *y*<*z* and similarly, *x*<*z* and *w*<*y*. A consequence of nonreversing X-junctions is that the luminance conditions are consistent with transparency and yet ambiguous with respect to depth ordering, and thus this form of X-junction supports two alternative transparency organizations. Although we have described the illusion as perceiving region *y* in the foreground, the X-junction also supports the alterative perception of region *x* in the foreground (in this case the likelihood of perceiving one or the other probably depends on the precise alignment of the contours in this natural scene).

**Figure 2.**
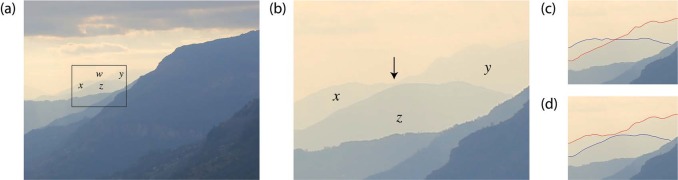
Transparent mountain illusion. (a) In the original image, mountain *y* is misperceived to be nearer in depth than mountain *x*, and area *z* appears to be a transparent region of overlap between the two. (b) In the enlarged image, it is clear that region *x* and *y* belong to the more distant mountain, and area *z* defines a nearer mountain. The arrow indicates the narrowest region in the photograph separating the two mountains, which leads to the misperception of a contour connecting the peaks defined by areas *y* and *z*, supporting the alternative (illusory) interpretation of transparency. The contour completion associated with the transparency illusion is outlined in (c); the veridically completed contours are outlined in (d).

The final classic principle from the history of perception involved in this illusion is the Gestalt principle of good continuation. The Gestalt psychologists emphasized the importance of grouping cues on visual perception. Wertheimer (1923) identified several specific grouping principles in his discussion of perceptual organization, including that of “good continuation.” The concept of good continuation is that contours appear to be connected by the shortest or smoothest route, i.e., they follow the “easiest” path. The contours involved in the illusion are outlined in Figure 2c and Figure 2d. Figure 2c shows how the contours are perceived under illusory conditions, as two relatively straight, overlapping lines. Figure 2d outlines the contours corresponding to the veridical interpretation of the scene, in which two lines come very close to touching in the middle, but then depart directions. According to the Gestalt principle of good continuation, the contours in Figure 2c (corresponding to the illusory interpretation) are more likely to be perceived than those in Figure 2d (corresponding to the veridical interpretation). The lines in the illusory case (Figure 2c) are also very similar to the classic example of good continuation as is demonstrated in many psychology textbooks.

In sum, the mountains appear transparent in this photograph not because of a Zen-like state induced by admiring a wonder of Nature, but because of three principles of visual perception that normally support veridical perception. In this scene, it is coincidental that the viewing angle and lighting elicit a set of conditions in which these perceptual inferences lead to misperception. The combination of appropriate luminance differences for perceptual transparency, in conjunction with physical contours that violate the principle of good continuation, leads to an incorrect segmentation of the scene, and in turn, a misperception of depth ordering. The reason this illusion is striking is that it relies on *both* the luminance conditions for transparency and the precise alignment of physical contours inconsistent with the principle of good continuation being present in the scene. As with most illusions, this example of an uncommon situation in which perceptual segmentation of the scene differs from the true, physical segmentation underscores how well the visual system normally integrates multiple segmentation cues to support veridical perception.
